# Anticoagulation in Emergency Cardiac Surgery — The Rationale for Modular Minimally Invasive Extracorporeal Circulation Use

**DOI:** 10.21470/1678-9741-2020-0481

**Published:** 2023

**Authors:** Ignazio Condello

**Affiliations:** 1 Department of Cardiac Surgery, Anthea Hospital, GVM Care & Research, Bari, Italy. E-mail: ignicondello@hotmail.it

The administration of heparin is crucial for cardiopulmonary bypass (CPB) establishment during cardiac arrest in an emergency cardiac surgery. Anticoagulation administration through an infusion line may not favor complete anticoagulation due to no and low flow during cardiopulmonary resuscitation, and an unreliable measure of preoperative activated clotting time (ACT) could expose the patient to the thrombotic risks of extracorporeal circuit and oxygenator during CPB initiation. Although there are guidelines for the management of anticoagulation during CPB, the topic of anticoagulation in emergency cardiac surgery is not exhaustively treated^[1]^. In normal conditions, heparin (300 U/kg) is administered intravenously before arterial cannulation with a target ACT > 480 seconds (measured after three minutes). During arterial cannulation, systolic pressure should be 90-100 mmHg to reduce the risk of aortic dissection. The aortic cannulation is done first to provide volume resuscitation in case of hypotension associated with venous cannulation. Once the aortic cannula is connected to the tubing, line pressure is checked to rule out dissection. After venous cannulation, venous clamp is gradually released to establish full CPB, and then ventilation is discontinued^[2]^. Due to the critical emergency conditions during cardiac arrest, extracorporeal membrane oxygenation (ECMO) was used as the primary option of choice to simplify the problems caused by anticoagulation; ACT testing is a non-specific point of care assessment method used since the 1970s when ECMO first started to be utilized. ACT is still widely used for dosage adjustment of the heparin drip in many ECMO centers. During heparin infusion, ACT levels should be kept between 180 and 220 seconds^[3]^. In the article “Low-Dose Heparin Anticoagulation During Extracorporeal Life Support for Acute Respiratory Distress Syndrome in Conscious Sheep”, by Prat NJ et al.^[4]^, a prospective cohort laboratory investigation was conducted to evaluate the coagulation function in an ovine model of oleic-acid induced acute respiratory distress syndrome supported with veno-venous ECMO. The experimental design approximated the time needed to transport from a battlefield setting to an advanced facility and compared bolus *vs*. standard heparin anticoagulation therapy. The study concludes that ECMO without anticoagulation may be a safe alternative in the early phase of trauma. However, despite being flexible for anticoagulant management, ECMO does not often have the components necessary to deal with an urgent cardiac surgery procedure. Relatively few published studies have examined these issues on CPB management in emergency cardiac surgery where the time factor is crucial for survival; in this context, I present the rationale for the use of modular minimally invasive extracorporeal circulation (MiECC). MiECC has been developed in an attempt to integrate all advances in CPB technology in one closed circuit that shows improved biocompatibility and minimizes the systemic detrimental effects of CPB. Despite well-evidenced clinical advantages, penetration of MiECC technology into clinical practice is hampered by concerns raised by perfusionists and surgeons regarding air handling together with blood and volume management during CPB^[5]^. The modular MiECC circuit, bearing an accessory circuit for immediate transition to an open system that can be used in every adult cardiac surgical procedure, offers enhanced safety features. An interesting study in a series of 50 consecutive patients, “Modular minimally invasive extracorporeal circulation systems; can they become the standard practice for performing cardiac surgery?” by Anastasiadis K. et al.^[5]^, shows that modular circuit design offers 100% technical success rate in a cohort of random, high-risk patients who underwent complex procedures, including reoperation and valve and aortic surgery, together with emergency cases. Since no evidence of increased thrombin formation was found from a laboratory standpoint, it was concluded that the use of MiECC with a reduced anticoagulation strategy seems possible. This alternative anticoagulation strategy leads to significant reduction in dosages of both heparin and protamine. We can confidently move forward with investigating this anticoagulation concept^[6]^. However, to establish clinical safety of ACT < 300 seconds, larger clinical studies are needed. From my point of view, modern MiECC systems are completely closed circuits containing a membrane oxygenator and a tip-to-tip surface coating that would allow to the operators in critical anticoagulant management to establish, in a first stage, the CPB with a closed system similar to ECMO management, and once the anticoagulation parameters have been ascertained and verified during the CPB, it’s possible to transform the system into conventional extracorporeal circulation management.

## References

[1] Shore-Lesserson L, Baker RA, Ferraris VA, Greilich PE, Fitzgerald D, Roman P (2018). The society of thoracic surgeons, the society of cardiovascular
anesthesiologists, and the American society of extracorporeal technology:
clinical practice guidelines-anticoagulation during cardiopulmonary
bypass. Ann Thorac Surg.

[2] Sarkar M, Prabhu V (2017). Basics of cardiopulmonary bypass. Indian J Anaesth.

[3] Kamdar A, Rintoul N, Raffini L (2018). Anticoagulation in neonatal ECMO. Semin Perinatol.

[4] Prat NJ, Meyer AD, Langer T, Montgomery RK, Parida BK, Batchinsky AI (2015). Low-dose heparin anticoagulation during extracorporeal life
support for acute respiratory distress syndrome in conscious
sheep. Shock.

[5] Anastasiadis K, Antonitsis P, Argiriadou H, Deliopoulos A, Grosomanidis V, Tossios P (2015). Modular minimally invasive extracorporeal circulation systems;
can they become the standard practice for performing cardiac
surgery?. Perfusion.

[6] Bauer A, Hausmann H, Schaarschmidt J, Szlapka M, Scharpenberg M, Eberle T (2019). Is 300 seconds ACT safe and efficient during MiECC
procedures?. Thorac Cardiovasc Surg.

